# Surgical repair of orocutaneous fistula in the left submandibular region of a day-old calf with ectopic incisors

**DOI:** 10.5455/javar.2022.i620

**Published:** 2022-09-30

**Authors:** Mohammad Raguib Munif

**Affiliations:** Department of Surgery and Obstetrics, Faculty of Veterinary Science, Bangladesh Agricultural University, Mymensingh, Bangladesh

**Keywords:** Congenital defects, orofacial fistula, abnormal incisors, reconstructive surgery, calf

## Abstract

**Objective:**

This study focuses on the orocutaneous fistula (OCF), a pathological channel between the buccal cavity and the outer surface (skin) of the face, causing leakage of saliva and fluid from the oral cavity to the face externally and also the ectopic incisors (EIs) erupted in a rare position in a day-old calf. The surgical interventions for these congenital defects were further described in this study.

**Materials and Methods:**

The calf was presented with an abnormal and external opening in the left submandibular region, having congenitally exposed jawbone and muscles with the resemblance of a linear groove, and was clinically examined based on the problem of swallowing milk due to lateral passing out of liquid through the unnatural tract before entering into the digestive tract. Clinical observation revealed an OCF with four EIs that abnormally erupted in the externally exposed groove of the fistula. Reconstructive surgery (RS) was performed along with a thorough guided approach to repair the defect, emphasizing normal functionality of the buccal cavity connecting to the pharynx and cranial esophagus, and smooth extraction of the EIs was ensured without making any deep wound.

**Results:**

After 2 weeks of postoperative care with supportive medications, the calf was quite recovered, having no further complications in the submandibular region along with no visible defect in swallowing.

**Conclusion:**

OCF in calves can be fruitfully treated by RS before getting severely infected and complicated. Outside the oral cavity, submandibular EIs can be easily removed, ensuring no further bleeding and defect.

## Introduction

A fistula is an abnormal passage or pathological opening either between two organs or vessels, between two hollow spaces (e.g., intestine), or between an organ and the surface of the body that are not connected normally [1]. Various types of acquired and congenital fistulae are reported in animals and humans, which include salivary, orocutaneous, nasal, oronasal, tracheal, tracheoesophageal, ruminal, rectovaginal, intestinal, perianal, urachal, urethral, and teat fistulae [2–7]. Orocutaneous fistula (OCF), often called an orofacial fistula, is a pathological communication between the oral cavity and the facial skin [8]. It is a very rare case to be found in calves. The most common causes considered for this fistula are traumatic and accidental injury, chronic dental infection, dental implant complications, salivary gland lesions, and malignancy [9,10], resulting in the abnormal flow of liquid, i.e., saliva, water, fluid, and other materials from the buccal cavity to the outer facial surface. Congenital fistulae are caused due to defects at birth by several genetic or environmental factors or a combination of both. Up to 5% of cases are found with such anomalies in calves, leading to various dysfunctions and death [6,11]. On the contrary, acquired fistulae are often the consequences of accidental injury and mechanical or sudden trauma from various objects [12]. There are few findings about the treatment protocols of OCF of facial origin in ruminants. The therapeutical options are often debatable and dependent on various factors, i.e., animal species, location and type of fistula, and duration of the untreated condition. In addition, different types of flaps and grafting techniques and conservative methods are conventionally used for the repair and reconstruction of fistulas [8]. Persistent and chronic fistulas are hard to deal with, and they cause a lot of pain and trouble during the whole process of getting the recovery.

Eruption of teeth is usually a localized and organized process that includes the complex phases of development of tooth buds, dental sockets, and remodeling of underlying bone and ramus at the proper time [13,14]. Various types of tooth eruption defects arise during the transitional period of dentition, including ectopic eruption that needs initial diagnosis and treatment to prevent more severe malocclusion and permanent loss of arch length [15]. Usually, ectopic dental eruption (EDE) takes place because of the deviation in the natural passage of tooth eruption from the apical to the distal surface, resulting in tooth lock [14]. EDE is more common in the maxillary region than in the mandible [16,17]. However, the actual etiology is controversial and sometimes multifactorial. To find this kind of defect, a clinical and radiographic diagnosis is recommended. If treatment is not done, it can lead to dental caries, severe localized pain, and other complications [14].

In animals, OCF, together with ectopic incisors (EIs), are very rare and peculiar cases to be found in field conditions. The treatments of these defects are not the usual practices that have been reported before. This case study has been performed to highlight the surgical management of the OCF and EIs that were clinically detected in the left submandibular region of a day-old calf.

## Materials and Methods

### Ethical approval

In this instance, ethical approval is not needed. This was a local case admitted to the Veterinary Teaching Hospital (VTH) of Bangladesh Agricultural University (BAU), Mymensingh-2202, Bangladesh, for treatment by registered veterinarians. The necessary documents for this case report were collected with the consent of the animal owner, including permission from the director of VTH, BAU.

### Case details

**Figure 1.**
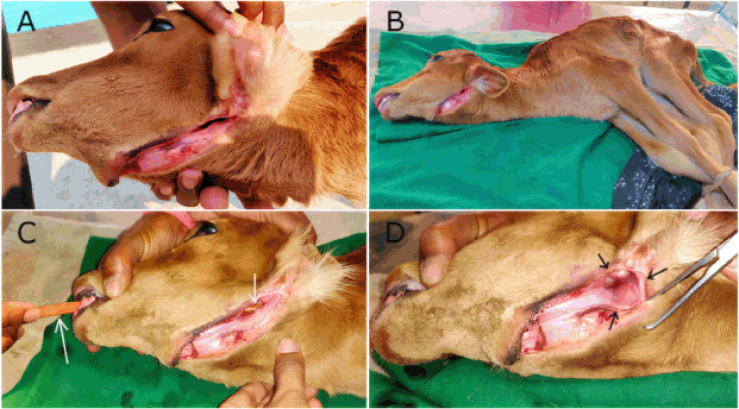
A day-old calf affected with OCF having clinical examination. (A) An exposed and linear passage in the left submandibular region, (B) physical restraining of the calf with upward exposure of the OCF, (C) intubation of stomach tube (white arrows) through mouth toward cranial esophagus, and (D) an oval-shaped opening (black arrows) below the angle of mandible.

A day-old calf of 24 kg body weight (BW) was referred to VTH of BAU in February 2022 with the complaint of a prominent external channel in the left submandibular region noted just after birth, causing hindrance to the swallowing process of milk (colostrum) and water. Severe struggle in swallowing was observed in the calf as fluid leaked laterally before arriving in the gastrointestinal tract (GIT) due to the spontaneous bypassing of liquid substances through the unusual lateral channel. After frequent manipulation and close inspection of the defect, a complete passage was found externally connected to the oral cavity internally. The passage was manifested as a linear, thick, and concave groove (Fig. 1A) having an oval-shaped hole just below the angle of the mandible, and the hole was fully opened into the buccal cavity. The abnormal groove had the appearance of an open wound and consisted of the body of a bony mandible in the middle with peripherally exposed muscle layers and skin. In addition, during wound exploration, a total of four abnormal incisor teeth were found, which had erupted within the wide groove, three together in one place with complete eruption and only one with an incomplete eruption in another place, being partially buried in the exposed jaw muscles. Eventually, the case was clinically diagnosed as a congenital OCF with four EIs in the calf with evidence of severe dehydration, discomfort, and weakness. No further radiography, computed tomography scan, or endoscopy of the oral cavity was performed. Finally, surgical repair and reconstruction were considered for dealing with the defects.

### Presurgical preparations

Before surgery, the calf was clinically monitored for physiological indices and was found to have a fairly normal temperature, heart rate, and respiratory rate. To check the existing dehydration, intravenous administration of 5% dextrose in normal saline (0.9% NaCl) was done to stabilize the animal. Then it was premedicated intramuscularly with Atropine Sulfate at 0.04 mg/kg BW (Atrovet^®^, Techno Drugs Ltd., Narsingdi, Bangladesh) followed by deep sedation with Xylazine HCl at 0.08 mg/kg BW (Xylaxin^®^, Indian Immunologicals Ltd., Hyderabad, India). Strict presurgical aseptic preparations were taken appropriately to ensure proper hygiene and avoid contamination.

### Reconstructive surgery (RS)

**Figure 2.**
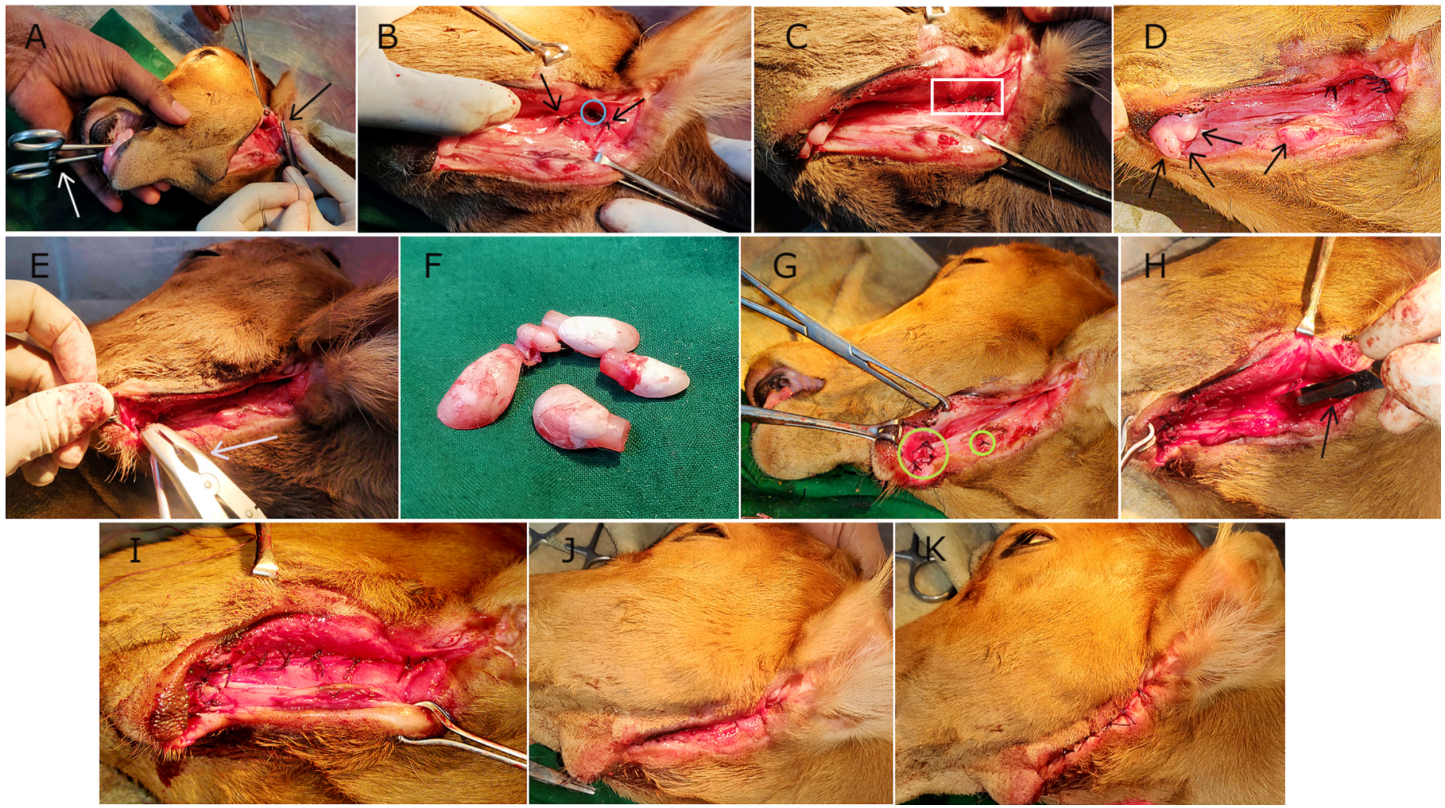
Various phases of RS for OCF complicated with EIs in the calf. (A) Insertion of curved intestinal forceps (white arrow) through mouth and suturing the hole (black arrow), (B) closure of the defect (blue circle) accompanied by two edge supporting stitches (black arrows), (C) second layer closure with interrupted sutures (white rectangle), (D) EIs (black arrows): three cranial and one caudal, (E) application of dental forceps (white arrow), (F) extracted EIs, (G) closure of dental sockets (yellow circles), (H) scratching of the linear tract with scalpel (black arrow), (I) initial closure of the tract, (J) closure with intradermal suture, and (K) final closure with interrupted sutures.

After proper preoperative procedures, the calf was placed in lateral recumbency on the operative table with the defective and exposed submandibular region upward (Fig. 1B). Surgical reconstruction of the OCF was carried out with the view to ensuring the vitality and patency of the normal passageway within the oral cavity, further communicating to the pharynx and cranial part of the esophagus, involving mainly the normograde flow of GIT. A smooth rubber tube (stomach tube) was used to pass through the mouth (Fig. 1C) of the sedated calf to guide the normal passage from the oral cavity to the esophagus. At first, the oval-shaped opening (Fig. 1D) directly connecting the oral mucosa to the external surface was considered. Light surgical scrapping of the mucosal wall was done, avoiding much bleeding to ensure a raw surface. In addition, a blunt and curved intestinal forceps was placed in the mouth to gently push the tongue ventrally and toward the floor of the buccal cavity for better exposure and to aid in closing the defective hole (Fig. 2A) with four stitches of interrupted sutures along with two edge-supporting stitches (Fig. 2B) using Polyglactin 910 of size 2-0 (Vicryl^TM^, Ethicon, J & J Medical Devices Companies, Irvine, CA). This was the initial closure, and again, a second layer of closure (Fig. 2C) with interrupted stitches was carried out over the first one to bury and reinforce it. The normal pressure, friction, and tension of that particular portion were highly considered during the repair process. Then the linear passage was surgically approached carefully as there were four EIs (Fig. 2D) found within the groove. The three cranial and one caudal abnormal EIs were carefully elevated and extracted by dental forceps (Fig. 2E and F), applying gentle twisting and traction. There was not much bleeding after dental extraction, and the unnatural dental sockets (muscular hollows) were flushed with normal saline to remove blood clots and thereafter closed routinely (Fig. 2G) with simple interrupted sutures using Vicryl^TM^ (2-0). After removing all EIs, the linear fistulous tract was scratched (Fig. 2H) gently to get raw tissues for effective closure and healing. Then the tract was initially closed with a single layer of simple interrupted sutures (Fig. 2I) using Vicryl^TM^ (1-0). In addition, a layer of intradermal suture was given (Fig. 2J) with chromic catgut of size 2-0 (Trugut^TM^, Sutures India Pvt. Ltd., Bangalore, India) to maintain the aesthetic view of the mandible. Lastly, the edges of the exposed skin were sewn together with braided silk in a simple interrupted pattern (Fig. 2K). This was done over the intradermal suture to give it more support and protection against pressure and movement, as well as to ensure better apposition of the uneven edges of the lesion.

### Postoperative care and management

**Figure 3.**
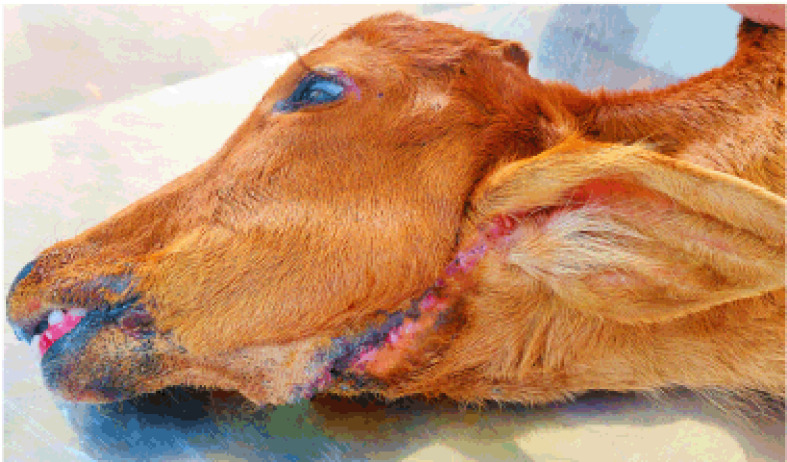
The calf after the removal of skin stitches on the 14th postoperative day.

Postoperatively, the calf was fully monitored for any further complications under the maintenance of essential supportive medications with intensive care. The supportive medicines included accurate courses of Ceftriaxone at 15 mg/kg BW (Triject Vet, Eskayef Pharmaceuticals Ltd., Dhaka, Bangladesh) at every 12 h interval for 14 days, Ketoprofen at 3.3 mg/kg BW (Ketovet, Techno Drugs Ltd., Narsingdi, Bangladesh) once daily for 5 days and Pheniramine Maleate at 1 mg/kg BW (Antihista-Vet^®^, Square Pharmaceuticals Ltd., Dhaka, Bangladesh) once daily for 7 days along with an intravenous infusion of 300 ml of 10% dextrose saline at every 12 h interval for the first 2 days after the operation. After 3 days, the calf started to swallow an adequate amount of milk, water, and other liquid substances without any loss. Oral vitamin-mineral supplements were provided from the 7th day after surgery for better healing and early recovery. The skin sutures were removed on the 14th postoperative day, and the calf was quite recovered (Fig. 3), having no further complications in the submandibular region along with no visible defect in swallowing. During the whole management period, fly repellents were used to prevent myiasis, and proper hygiene of the animal shed was ensured.

## Results and Discussion

In this study, the OCF and the EIs of the 1-day-old calf were of congenital origin. Various types of congenital defects in calves have been reported [18,19], although few of them belong to the facial and oral regions. Congenital craniofacial defects have been observed in calves [20], whereas orocutaneous, oromaxillary, and oronasal fistulae have been found in different species [21,22]. Likewise, EDE and abnormal teeth have been reported previously [23,24]. In this study, the ectopic eruption of incisor teeth was noted, which is almost rare in bovine calves as a congenital disorder.

The calf in this study was found dehydrated and emaciated, which might be attributed tothe partial loss in milk consumption due to the defect in swallowing from birth. To the best of knowledge, this type of defect has not yet been reported in day-old calves, where OCF along with congenital EIs combinedly made an abnormal case.

The calf was treated with RS to confine the fistula after ascertaining the normal swallowing process without any defect. Various types of RS have been performed previously to correct a variety of fistulae and other defects of facial origin [7,8,18,25]. Different types of flaps such as rotational, trans-positional, autogenous, alloplastic, deltopectoral, bony alveolar, mucoperiosteal, and glandular pedicled flaps can be grafted successfully for surgical repair and reconstruction of OCF [2,8,25,26]. Besides, non-conventional techniques and diversified approaches other than tissue grafting for the management of numerous fistulae of the oral cavity have been reported by many researchers [27–30]. On the other hand, there are several conservative treatment protocols such as reducing salivary secretions by supportive medications, nasogastric tube feeding, packing, and vacuum-assisted closure to handle the OCF without severe complications [8,21]. However, the present RS of the calf mainly included the guided approach for corrective closure of the defective mucosal wall along with exposed muscle layers encompassing the parts of the left mandibular angle and body with several layers of sutures. This type of closure was feasible for the correction of the OCF because it was not too difficult to close the entire fistulous tract along with the cavity-surface connecting opening by the discussed technique, and there was no need for extra use of tissue grafting, i.e., flap techniques or other device-assisted protocols. This was possible due to no severe deformity and agenesis in the bony mandible as well as in the oral mucosa, fascial muscle, and skin of the calf.

The EIs were conveniently removed during the manipulation and surgical debridement of the fistula before successful closure. The abnormal incisors were surgically extracted by dental forceps, emphasizing not making deep cavities within the groove of the fistula. Several authors [31–33], reported the extraction of a variety of unwanted teeth erupted in a faulty manner, causing troubles in various species. The removal of the abnormal incisors was thought to be comparatively easier with gentle torsion and lifting, as they were freely embedded in the chambers of tough muscle fibers, not as typical as normal teeth seated into the alveolar bony socket with the attachment of the periodontal ligaments and prominent vessels. Hence, there was very little or no bleeding during the extraction process, and the holes developed after the dental withdrawal were securely sealed up by suturing. This was possible due to the presence of adequate muscle flaps without bony structures.

Suturing was performed with absorbable suture materials, and double-layered sutures were applied in the fistulous tract with the simple interrupted pattern to sustain against excess pressure, motion, and rotation imposed on the jaws during daily oral function.

In this study, Atropine Sulfate and Xylazine HCl were used for premedication and sedation of the calf before the surgery, which is correlated with the findings of other researchers [34,35]. The possible differential diagnosis includes a wide range of localized skin infections and osteomyelitis, periodontal infections, sweat gland occlusion, actinomycosis, tuberculosis, congenital midline sinus of the hard palate and upper lip, pyogenic granuloma, odontogenic cysts and tumors, salivary gland infections and neoplasms (benign or malignant), and various pathologic conditions of the buccal cavity [36–38]. OCFs are often found secondary to the surgery of oral squamous cell carcinoma [39], and laryngectomy and other oral cavity surgeries might provoke this defect [40]. In contrast, EIs are more often associated with genetic defects than any sequel of oral surgeries.

There might be chances of infections, recurrence, and tearing of suture lines after surgery. However, this study did not observe these types of postoperative complications, which might be attributed to the efficiency of surgical techniques and hygienic measures along with supportive postoperative medications.

## Conclusion

Congenital OCF in calves located in the submandibular region can be repaired effectively by RS. EIs in day-old calves can be fruitfully handled by smooth dental extraction and sealing of the chambers. Postoperatively, good management with intensive care is the precondition for better healing and recovery.

## List of abbreviations

BAU, Bangladesh Agricultural University; BW, body weight; EDE, ectopic dental eruption; EIs, ectopic incisors; GIT, gastrointestinal tract; OCF, orocutaneous fistula; RS, reconstructive surgery; VTH, Veterinary Teaching Hospital.
